# Biochemical Discrimination between Selenium and Sulfur 1: A Single Residue Provides Selenium Specificity to Human Selenocysteine Lyase

**DOI:** 10.1371/journal.pone.0030581

**Published:** 2012-01-25

**Authors:** Ruairi Collins, Ann-Louise Johansson, Tobias Karlberg, Natalia Markova, Susanne van den Berg, Kenneth Olesen, Martin Hammarström, Alex Flores, Herwig Schüler, Lovisa Holmberg Schiavone, Peter Brzezinski, Elias S. J. Arnér, Martin Högbom

**Affiliations:** 1 Structural Genomics Consortium, Department of Medical Biochemistry and Biophysics, Karolinska Institute, Stockholm, Sweden; 2 Stockholm Center for Biomembrane Research, Department of Biochemistry and Biophysics, Arrhenius Laboratories for Natural Sciences C4, Stockholm University, Stockholm, Sweden; 3 Division of Biochemistry, Department of Medical Biochemistry and Biophysics, Karolinska Institutet, Stockholm, Sweden; University of Queensland, Australia

## Abstract

Selenium and sulfur are two closely related basic elements utilized in nature for a vast array of biochemical reactions. While toxic at higher concentrations, selenium is an essential trace element incorporated into selenoproteins as selenocysteine (Sec), the selenium analogue of cysteine (Cys). Sec lyases (SCLs) and Cys desulfurases (CDs) catalyze the removal of selenium or sulfur from Sec or Cys and generally act on both substrates. In contrast, human SCL (hSCL) is specific for Sec although the only difference between Sec and Cys is the identity of a single atom. The chemical basis of this selenium-over-sulfur discrimination is not understood. Here we describe the X-ray crystal structure of hSCL and identify Asp146 as the key residue that provides the Sec specificity. A D146K variant resulted in loss of Sec specificity and appearance of CD activity. A dynamic active site segment also provides the structural prerequisites for direct product delivery of selenide produced by Sec cleavage, thus avoiding release of reactive selenide species into the cell. We thus here define a molecular determinant for enzymatic specificity discrimination between a single selenium versus sulfur atom, elements with very similar chemical properties. Our findings thus provide molecular insights into a key level of control in human selenium and selenoprotein turnover and metabolism.

## Introduction

As an essential trace element, selenium is incorporated into selenoproteins in the form of selenocysteine (Sec), the selenium analogue of cysteine (Cys), by a co-translational process redefining specific UGA codons thereby expanding the genetic code [1], [2]. The human genome contains 25 known genes encoding selenoproteins some of which are essential for mammals [3]. In spite of its important role as the defining entity of selenoproteins, selenium can also be severely toxic because of high chemical reactivity of metabolites such as selenite and hydrogen selenide [4], [5], [6]. Thus it becomes crucial for selenoprotein-dependent organisms to have adequate selenium intake as well as to develop means for tight control of the selenium metabolism. One unique property in synthesis of Sec, likely developed as a means of controlling its reactivity, is the fact that Sec, in contrast to the 20 other common amino acids, is not loaded as such onto its cognate tRNA but is instead synthesized directly on tRNA^Sec^. This tRNA species is initially amino acylated with a seryl residue, tRNA^Sec[Ser]^, which in archaea and eukaryotes is converted to tRNA^Sec[Sec]^ *via* an O-phosphoseryl-tRNA^Sec[Ser]^ intermediate by Sec synthase, utilizing selenophosphate as the selenium donor [7], [8], [9]. Recently the structural basis for this step was revealed through studies of a crystal structure of the human SepSecS-tRNA^Sec^ complex [10]. Selenophosphate is provided by at least one isoform of selenophosphate synthetase (SPS), utilizing selenide and ATP [8]. The source of selenide for that reaction may derive from either selenite reduction, in a reaction that may be catalyzed by thioredoxin reductase [11], from conversion of methylated low molecular weight selenium compounds [12], or through selenium removal from Sec as catalyzed by Sec lyase (SCL) [13], [14], [15], [16]. Sec as a precursor for the latter reaction should derive from all events of selenoprotein degradation. This path may be of particular importance for the high selenium retention in brain or in other tissues where selenoprotein synthesis is dependent upon selenium derived from degradation of selenoprotein P [5], [17]. If free Sec would be released from selenoprotein degradation it should easily be detrimental to a cell due to its inherent chemical reactivity and Sec catabolism must therefore be tightly controlled. The Sec degradation step, as catalyzed by SCL, can thus be expected to be regulated and of high capacity. Interestingly, human SCL (hSCL) is unlike several bacterial orthologues specific for Sec and does not accept the sulfur analogue Cys as substrate with any appreciable activity. Instead of displaying Cys desulfurase (CD) activity, hSCL is even inhibited by addition of excess Cys [13], [18], [19], [20]. Sequence similarity analyses and available biochemical data [13], [16] suggest that the presence of a SCL specific for Sec is a feature of higher eukaryotes, well in line with the need for a strict control of selenium metabolism in these selenoprotein-dependent organisms. SCL from pig was the first enzyme discovered to act specifically on selenium compounds [13].

SCL and CD enzymes are NifS-like proteins that contain a completely conserved active site Cys residue (C388, hSCL numbering) that form an enzyme-bound persulfide (Cys-S-SH) or sulfoselenide (Cys-S-Se^−^) as product of the reactions with Cys or Sec, respectively [21]. The (Cys-S-Se^−^) species is commonly also denoted “perselenide” in the literature but here sulfoselenide is used to avoid confusion with a Sec-Se-Se^−^ perselenide. NifS proteins can be divided into two major groups (groups I and II) based on sequence characteristics [22], but enzymes of lower organisms from either group were shown to have both SCL and CD activity [20], [22]. Group-I proteins, to which the SCLs of higher eukaryotes belong, contain a 10- to 12-residue insertion compared to group II. This produces a larger segment encompassing the active site Cys that is conserved in all enzymes from both groups. In structures of group-I enzymes, this segment is commonly disordered and not visible in the electron density.

During recent years it has become clear that persulfides serve as the predominant sulfur donors in synthesis of sulfur-containing biomolecules and that enzymes involved in metabolism of sulfur compounds often form protein-protein complexes to traffic or deliver the sulfur atom [23], [24], [25]. Because of the relative rarity of selenium compared to sulfur, its higher chemical reactivity and its toxicity, defined and controlled trafficking events for selenium are expected to be even more important than for sulfur. This imposes a challenging task on organisms, considering the close similarities between sulfur and selenium in terms of chemical properties. Though less studied than their counterparts involved in sulfur metabolism, a body of data is emerging indicating that eukaryotic SCL proteins, as well as their bacterial orthologues, may be involved not only in selenium assimilation and Sec recycling, but also in controlled delivery of selenium for selenophosphate synthesis [16], [19], [26], [27], [28], [29], [30], [31].

The structural and chemical basis for the important and strict selenium specificity of eukaryotic SCLs remains unclear. A recent study of a SCL from rat (rSCL) crystal structure reports slightly different binding modes for Cys and selenopropionate, used as a Sec substrate analogue, suggesting this to be the basis for specificity [32]. In the same study, Cys was found to reversibly form a nonproductive adduct with rSCL while selenopropionate bound in two different conformations [32]. The specific production of a sulfoselenide adduct to the active site Cys residue was also shown [32]. However, the guiding mechanism for the substrates, and whether the lack of an amine on the Sec substrate analogue that was used influenced its binding remains an open question. Here, we present the crystal structure of hSCL, which helps resolve this question. Using a structure-guided bioinformatic approach, a single Asp residue that confers Sec specificity was identified and we could subsequently design variant proteins that gained CD activity in addition to having maintained SCL activity, thus defining a molecular determinant for Sec specificity of the wild-type enzyme. The structure also revealed the features of the dynamic active site segment including the conserved C388, suggested to allow control and delivery of the selenide produced in the Sec lyase reaction.

## Results

### Overall structure

**Figure 1:**
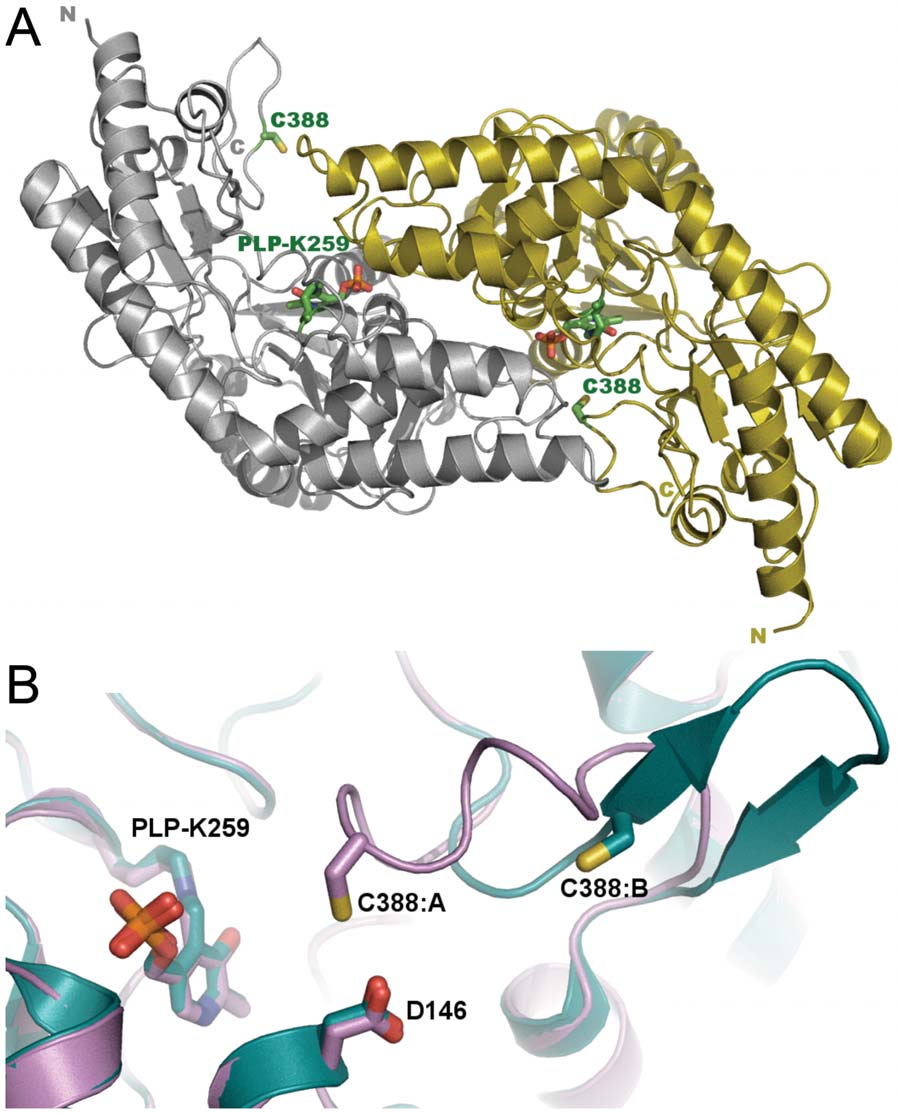
Structure of Human SCL. A) Human SCL homodimer in complex with the co-factor PLP. The C388 residues of both subunits are indicated. B) Superposed subunits A and B of human SCL showing the structural differences in the active site segment and positioning of C388 (subunit A: pink “closed” and subunit B: cyan “open”), the location of Asp 146 is also shown.

Structures of hSCL in two different crystal forms were determined: P1 to 1.8 Å, with four monomers per asymmetric unit and P2_1_2_1_2_1_ to 2.1 Å resolution, with two monomers per asymmetric unit (Table 1). Similar to the non-specific SCL/CD enzymes, hSCL was found to adopt the canonical fold type I structure of PLP enzymes [33], forming two active sites in the homodimer interface with each monomer contributing residues to both active sites (Fig. 1A).

**Table 1:** Data collection and refinement statistics.

	Structure 1	Structure 2
Data collection*		
Space group	P2_1_2_1_2_1_	P1
Cell dimensions		
a, b, c (Å)	59.2, 85.8, 188.6	66.6, 72.2, 89.4
a, b, g (°)	90, 90, 90	83.9, 68.4, 87.0
Resolution (Å)	20.0-2.10 (2.20-2.10)	30.0-1.80 (1.90-1.80)
Rsym	9.9 (34.4)	5.1 (52.7)
I/sI	13.1 (5.7)	16.2 (2.2)
Completeness (%)	99.8 (100.0)	94.1 (93.5)
Redundancy	7.1 (7.3)	2.0 (2.0)
Wilson B-factor	36.0	22.5
Refinement		
Resolution (Å)	20.0-2.10	30.0-1.80
No. reflections	56897	134466
Rwork/Rfree	0.182/0.235	0.184/0.217
No. atoms		
Protein	6197	12210
Ligand/ion	30	76
Water	457	899
B-factors		
Protein	33.2	15.3
Ligand/ion	27.5	23.6
Water	36.0	27.8
R.m.s. deviations		
Bond lengths (Å)	0.018	0.014
Bond angles (°)	1.70	1.46
PDB accession code	3GZC	3GZD

One crystal was used for each structure.

*Values in parentheses are for highest-resolution shell.

### Structure of the segment containing the conserved active site cysteine

**Figure 2:**
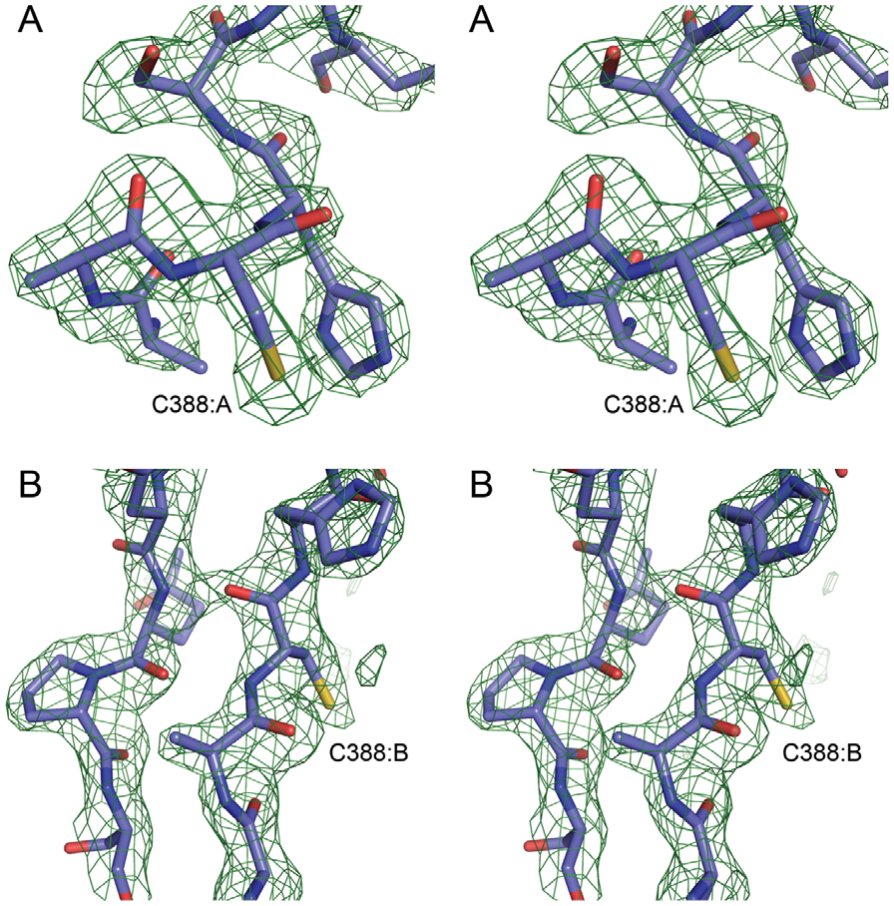
Electron density for the dynamic segment. A and B) Stereo figure of an Fo-Fc difference electron density omit map of the segment surrounding C388 for chains A and B, contoured at 2.5σ (0.11 eÅ-3).

In the P2_1_2_1_2_1_ structure, the segment containing C388 (residues 385–396) was ordered in both subunits of the asymmetric unit. Interestingly, it adopted two different ordered conformations in the two monomers. In subunit A, the segment was folded onto the protein in a near α-helical “closed” conformation, restricting access to the active site and placing the active site C388 in proximity to the PLP cofactor. In contrast, the corresponding active site segment of subunit B displayed an extended β-hairpin structure (Fig. 1B), positioning C388 more than 17 Å from the PLP cofactor and exposing it to solvent. An electron density omit map for the segment surrounding C388 in monomers A and B is shown in stereo figures 2A and B respectively.

**Figure 3:**
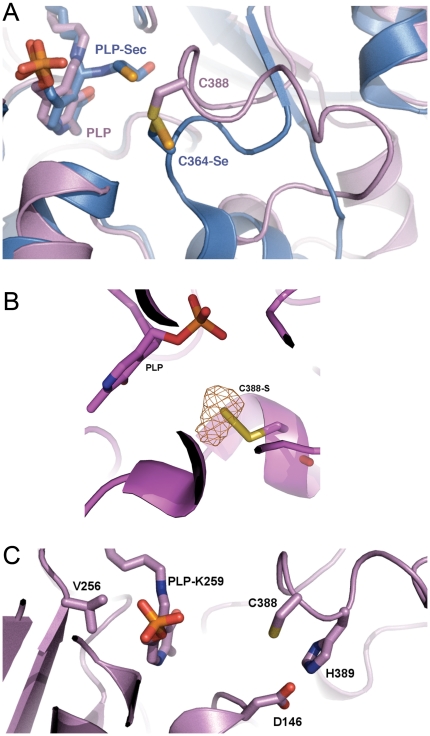
Active site. A) Human SCL in pink showing the PLP binding site and *E. coli* NifS/CsdB (PDB: 1KMK) with the Cys-sulfoselenide intermediate in blue showing the similar positioning of the C388-sulfur atom (hSCL) and that of C364 in *E. coli* NifS/CsdB. A second Sec substrate molecule, bound to the PLP in the E. coli NifS/CsdB [21], is also shown. B) Electron density for the C388 persulfide in subunit A after a 2 h Cys soak of a P1 spacegroup hSCL crystal. Fo-Fc difference electron density map of the C388 persulfide with the δ-sulfur omitted contoured at 4σ (0.23 eÅ-3). C) Position of mutated residues D146K, V256S and H389T in relation to C388 and the PLP cofactor.

The peptide backbone of the dynamic segment containing the conserved active site Cys did not align in space with its counterpart found in available structures of group-II NifS-like proteins. Remarkably, in spite of the completely different backbone architecture, the closed conformation of hSCL positioned the C388 sulfur atom in an identical position in relation to the PLP cofactor and substrate-binding site as observed in the group-II proteins. In the *E. coli* group-II NifS protein, both persulfide and sulfoselenide intermediates have been shown to be produced at the active site cysteine with this particular reaction geometry [21] (Fig. 3A). Moreover, in a second crystal form (P1) of hSCL, crystallized at a higher pH of 8.1 and soaked with 10 mM Cys for 2 hours, the electron density of subunit A showed the formation of an adduct, most likely a C388-persulfide, to C388 in the closed conformation (Fig. 3B). The persulfide adduct was present in subunit A with near full occupancy and also in subunit D, but there at lower occupancy. Together, this strongly suggests that the closed conformation observed in the present structure is involved in catalysis and C388-sulfoselenide formation using Sec as a substrate. The formation of a C388-persulfide after 2 hr incubation with 10 mM Cys is also an important clue to the chemical basis for the inherent Sec specificity of the enzyme because it indicates that hSCL is not, at least under these crystallization conditions, absolutely specific for Sec but may perform Cys desulfurization with extensive concentrations of Cys over very long time scales. However, this should have no biological relevance since the reaction rate is insignificant in activity assays [13], [16], [18]. It should be noted that although the dynamic segment (residues 385–396) was visible in the electron density in both subunits in the P2_1_2_1_2_1_ structure it seemed less rigid than the rest of the protein, as indicated by higher B-factors and a somewhat weaker electron density. In particular the density for Gly 393 of subunit B was poor. The inherent dynamic property of this segment was also confirmed by the structure in the P1 crystal form where, similar to structures of other Group-I enzymes, it was disordered to different degrees in all four monomers of the asymmetric unit. Residues A 391–394, B 386–394, C 389–393 and D 391–394 are missing from the electron density in this crystal. It appears that the dynamics of this particular protein segment is a common feature among group-I NifS-like proteins.

### Gain-of-function substitutions

**Figure 4:**
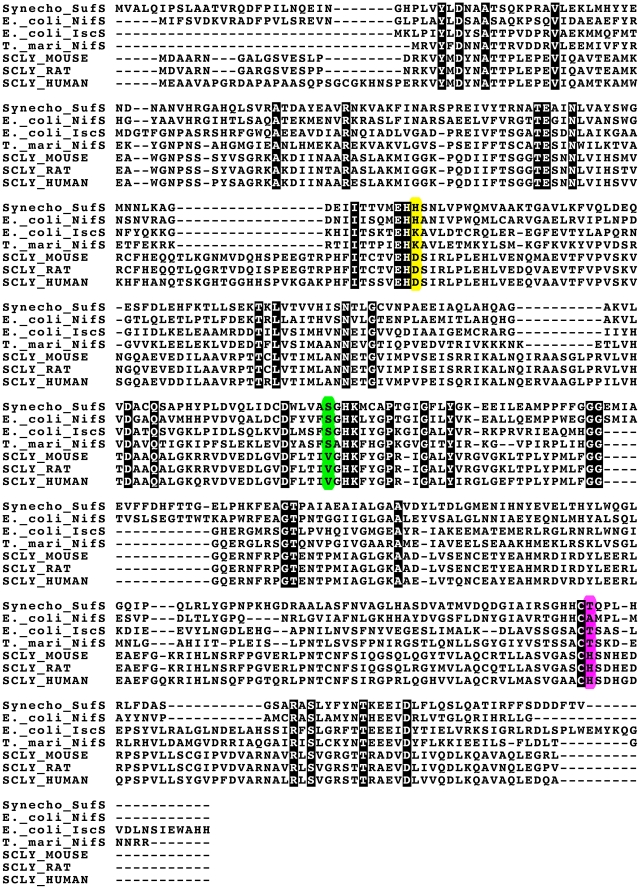
Alignment of representative sequences of bacterial SCL/CD enzymes (*Synechocystis* SufS, *E. coli* NifS, *E. coli* IscS and *T. maritima* NifS) and Mammalian Sec-specific SCLs (Mouse, Rat and Human). Residue positions corresponding to D146, V256 and H389 (hSCL numbering) are indicated with a yellow, green and purple background respectively.

**Figure 5:**
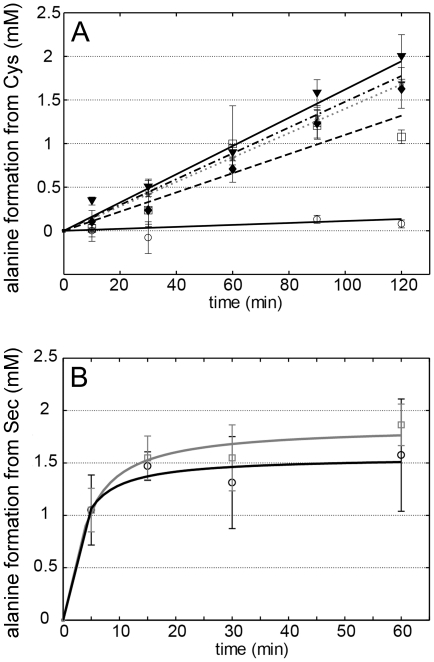
Activity of wild-type and variant proteins. A) Activity of WT hSCL and variants containing the D146K substitution with Cys. WT hSCL, solid black line and open circles; D146K/V256S/H389T, dotted grey line and closed diamonds; D146K, dot-dash black line and stars; D146K/V256S, dash black line and open squares; D146K/H389T, solid black line and filled triangles. B) Activity of WT hSCL and the D146K/H389T variant with Sec. WT, black line and open circles; D146K/H389T variant, grey line and open squares.

To be able to study the chemical basis for Sec specificity, we endeavored to produce gain-of-function variant proteins with acquired CD activity. Based on the hSCL structure and sequence alignments between non-specific and Sec-specific enzymes, we identified three residues in the active site vicinity that could be thought to contribute to the Sec specificity (Fig. 3C and Fig. 4). Two of the residues (D146 and H389) are positioned less than 4 Å from C388, essentially in direct van der Waals contact. Residues corresponding to D146 are conserved as Asp in higher eukaryotes but are found as Lys in other Group-I proteins or as His in Group-II proteins (Fig. 4, yellow). Residues corresponding to H389 are conserved as His in higher eukaryotes, but found as Thr or Ala in non-specific proteins (Fig. 4, purple). The third selected residue (V256) is in van der Waals contact with the PLP cofactor. This residue is conserved as Val in higher eukaryotes but replaced by Ser (or in a few cases Thr) in other organisms (Fig. 4, green). Based on these evolutionary clues we generated D146K, H389T and V256S variants and we expressed, purified and analyzed the seven possible combinations of these hSCL substitutions. Activity assays revealed that a novel CD activity was obtained for all of the variants containing the D146K substitution (Fig. 5A). In the absence of the D146K substitution the other substitutions did not confer detectable activity with Cys. The activity with Sec observed in the wild-type enzyme was not altered within experimental error in the D146K/H389T variant (Fig. 5B) and the initial rate was several-fold higher than with Cys for the variant enzymes (Fig. 5A). As both substrates were initially present at an equivalent of 10 mM, the reduction in activity (curvature) in the reaction with Sec after ≈10 minutes is likely due to some form of product inhibition or depletion of the Sec substrate by a competing non-enzymatic reaction.

## Discussion

### Gain-of-function for cysteine desulfurase activity

By using a structure-guided bioinformatic approach we were here able to identify a number of residues that potentially could influence the substrate specificity of SCL enzymes. Among these variants, change of the single Asp146 residue to Lys proved necessary and sufficient to achieve CD functionality in hSCL. Double and triple mutants, including the D146K variation, all showed CD activity. The other variations, H389T and V256S, influenced the activity only slightly when combined with the D146K variation but did not yield any detectable CD activity in the absence of the D146K variation. These results are consistent with available specificity data for SCL/CD enzymes and should allow for more robust bioinformatic assignments of Sec/Cys specificity in SCL/CD enzymes. The residue that, upon charge inversion, confers gain-of-function for CD activity is located close to C388, but distant from both the PLP cofactor and the substrate-binding site. This could suggest that the chemical properties of C388 are directly involved in the observed specificity of hSCL. A study of the mechanistic implications of the D146K change is described in an accompanying paper [34].

Previous studies of hSCL have reported K_m_ of 0.5 mM with Sec and a K_i_ for Cys of 5.85 mM [18]. The k_cat_ for mouse SCL with Sec has been reported as 46 s^−1^ with a K_m_ of 9.9 mM and in that study catalytic parameters for Cys as the substrate was also reported with a k_cat_ of 0.0058 s^−1^ and a K_m_ of 5.2 mM [16]. These numbers for K_m_ may appear high considering the expected low concentration of Sec substrate in the cell, possibly suggesting the importance of protein-protein interactions during delivery of substrates to SCL. Still, K_m_ is a kinetic parameter and not directly related to the dissociation constant K_d_. The high K_i_ value for Cys would also indicate that hSCL is not significantly inhibited by free Cys at any physiological concentrations. Future studies of hSCL activities in a cellular context are needed to further address these questions and the role(s) of hSCL *in vivo*. In the present study we have reached novel insights into the structural determinants for its activity and enzymatic properties.

### Possibilities for product protection and delivery

The reaction of SCL/CD enzymes with Sec results in a selenium-charged enzyme in the form of an enzyme-bound Cys-sulfoselenide [20], [21], [22], [32]. Interestingly, in group-I enzymes, this cysteine (C388 hSCL numbering) appears universally located in a dynamic segment, shown to be able to adopt at least two ordered states in hSCL (this work). In addition, a disordered state was observed in the P1 crystal of hSCL, as previously described also for other group I enzymes.

**Figure 6:**
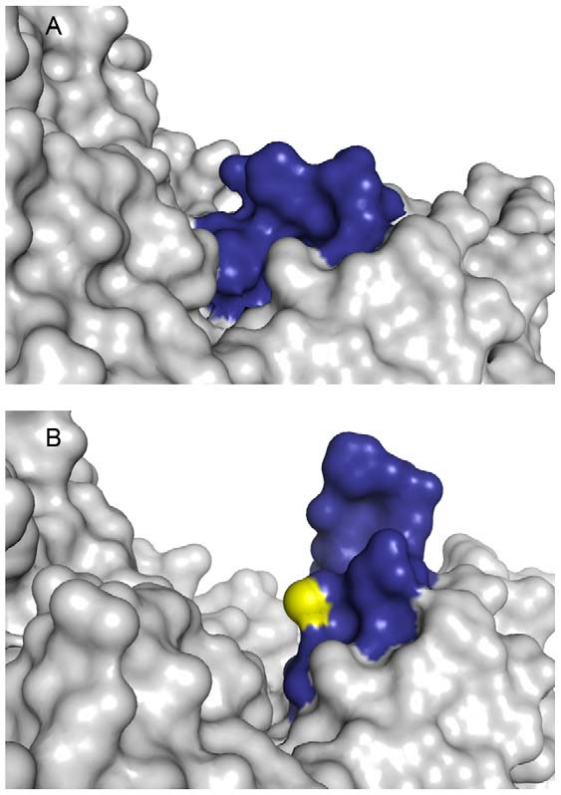
Conformations of the active site dynamic segment (blue). A) Closed, B) Open. The sulfur atom of C388 is shown in yellow.

**Figure 7:**
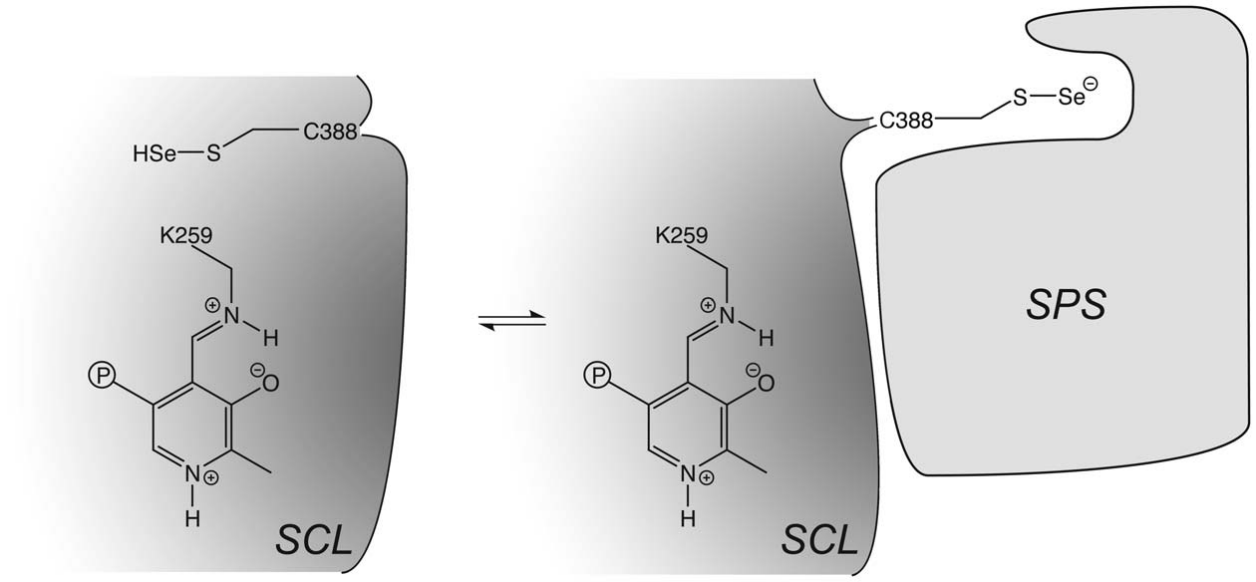
Potential mechanism of selenide product delivery directly to selenophosphate synthetase by the C388-containing flexible active site segment.

The hSCL structure shows that in the closed state, relevant for sulfoselenide formation, the reactive sulfur atom of C388 is buried in the protein and completely covered by the residues of the dynamic segment (Fig. 6A). However, in the open form, the sulfur atom is exposed and accessible to solvent (Fig 6B). It thus appers possible that the closed conformation can function not only in catalysis to support the sulfoselenide formation, but also as a storage conformation preventing the sulfoselenide to react unspecifically with surrounding cellular components. Possibly, interaction of SCL with selenophosphate synthetase (SPS), the enzyme in the Sec synthesis pathway using selenide as substrate, may trigger opening of the dynamic segment in SCL allowing direct delivery of selenide for SPS catlysis (Fig. 6B and Fig. 7). Several studies have indeed shown that NifS-like proteins can mobilize selenium from Sec in order for the selenide to act as a substrate for selenophosphate synthesis by SPS [19], [26], [27], [30]. SPS proteins contain a residue, close to the N-terminus, that is conserved as either Cys or Sec. That residue, C17 in *E. coli* SPS, has been suggested as a site accepting selenide in the wild-type protein, based upon isotope labeling experiments [35] and also supported by a recent structure of the *Aquiflex aeolicus* SPS [36]. Interestingly, only one of the two SPS homologs present in human, hSPS2, contain the corresponding Sec residue while hSPS1 contain a threonine in the corresponding position. Biochemical studies suggest that hSPS2 is used for selenite assimilation while hSPS1 is used for recycling of Sec, likely via the action of SCL [30]. An interesting observation in this context is that the *E. coli* SPS protein C17S variant is completely inactive in the standard in vitro assay with selenide as a substrate. In contrast, if selenium is provided as Sec together with any of the three *E. coli* NifS homologs (that all have SCL activity) the activity is partly restored [27]. In light of these results it is possible to suggest that the Sec/Cys residue in SPS may be used to capture a freely diffusing selenide substrate and deliver it to the active site. However, if the selenium would be provided in the form of a Cys sulfoselenide in the flexible active site segment of SCL, it could be delivered directly to the SPS active site via a SCL-SPS protein-protein interaction. The SCL Cys sulfoselenide would then replace delivery by the intramolecular Sec/Cys containing flexible loop in SPS (Fig. 7). In this context it is interesting to note that a recent study showed coimmunoprecipitation of the SCL and both SPS proteins from mouse [37]. However, other studies suggest that SPS1 does not play a direct role in selenocysteine metabolism [38], [39]. Further studies are clearly needed to firmly establish these complicated pathways.

### Conclusions

The chalcogen group of the periodic table provides no less than three elements that are incorporated into biological macromolecules: oxygen, sulfur and selenium. As one mechanism of control in selenium metabolism, higher eukaryotes have developed SCL proteins that are specific for Sec. When analyzing a set of variants of hSCL, a single D146K substitution was here found to be both necessary and sufficient to obtain CD activity and lose Sec specificity. Thus we have defined a molecular determinant that conveys selenium specificity to human SCL. Interestingly a single amino acid residue, not directly participating in catalysis, was found to provide this specificity. hSCL is thus a salient example of how more stringent chemistry can be evolved in certain enzymes of a larger group while keeping the same overall structure, cofactor and active site architecture. We also found that the structural properties of the active site dynamic segment of hSCL are well suited for control and delivery of selenium. It seems reasonable that these elegant features of the enzyme act together as a means to avoid release of toxic Se-species in cells during turnover and synthesis of selenoproteins.

## Materials and Methods

### Cloning and protein expression

The hSCL cDNA clone was purchased from the Mammalian Gene Collection (Genebank accession no. BC007891.2). The sequence encoding residues 8–445 was amplified by PCR using primers “forward TACTTCCAATCCATGGGGAGGGATGCGCCGG”, and “reverse TATCCACCTTTACTGTCAGGCCTGGTCCTCCAGCTG”) and inserted into pNIC-Bsa4 vector (Genbank accession no. EF198106) by ligation independent cloning. The construct included an N-terminal tag consisting of a 6-His sequence separated from the hSCL sequence by a TEV protease cleavage site. For expression the pNIC-Bsa4 containing the insert was transformed into the *E. coli* BL21(DE3) strain and stored at −80°C as a glycerol stock. Cells from the glycerol stock were used to inoculate 20 ml of Terrific Broth (TB) supplemented with 8 g/l glycerol and 100 µg/ml kanamycin and grown at 30°C over night. The 20 ml culture was used to inoculate 1.5 l TB media supplemented with 8 g/l glycerol, 50 µg/ml kanamycin and 5 drops of BREOX anti-foaming agent (Cognis Performance Chemicals UK Ltd) in a 2 l glass flask. Cells were grown in a Large Scale Expression System (LEX, Harbinger Biotechnology) at 37°C until the OD_600_ reached 1. The cultivation was then cooled to 18°C for 1 h whereupon expression of hSCL was induced by the addition of 0.5 mM IPTG and subsequently continued over night at 18°C. Cells were harvested by centrifugation at 5500× g for 10 min. at 4°C and the pellet was resuspended in lysis buffer containing 50 mM Na-phosphate pH 7.5, 500 mM NaCl, 10% glycerol, 10 mM imidazole, 0.5 mM TCEP, and Complete EDTA-free protease inhibitor (Roche Biosciences). Resuspended cells were stored at −80°C.

### Protein purification

The frozen cell suspension was thawed and 4 µl of 250 U/µl benzonase (Novagen) was added per 50 ml of suspension. The sample was sonicated on ice (Sonics VibraCell) at 80% amplitude, 4 sec on, 4 sec off for a total of 3 min followed by centrifugation at 49 000× g for 20 min. at 4°C. The soluble fraction was decanted and filtered through a 0.45 µm filter.

Purification of the protein was performed as a two-step process on an ÄKTAxpress system (GE Helthcare). First step, metal affinity chromatography using 1 ml HiTrap Chelating column (GE Helthcare) and second step, gel filtration using, Superdex 200 gel filtration column (HiLoad 16/60; GE Healthcare). Prior to purification, the HiTrap Chelating column was equilibrated with *buffer1* (50 mM Na-phosphate pH 7.5, 500 mM NaCl, 10% glycerol, 10 mM imidazole, 0.5 mM TCEP) and the Superdex 200 was equilibrated with *buffer2* (20 mM Hepes pH 7.5, 300 mM NaCl, 10% glycerol, 0.5 mM TCEP. The filtered lysate was loaded onto the Ni-charged HiTrap Chelating column and washed with *buffer1* followed by *buffer1* supplemented with imidazole to a final concentration of 25 mM. Bound protein was eluted from the column with *buffer1* containing 500 mM imidazole and automatically loaded onto the gel filtration column, and subsequently eluted using *buffer2*. The UV_280_ absorption chromatogram of the eluate showed a single major peak at a retention volume of 74 ml. This peak consisted of hSCL as analyzed by SDS-PAGE and the fractions from the peak were colored bright yellow from the bound PLP cofactor. Fresh TCEP was added to the pooled fractions to a final concentration of 2 mM and the protein was then concentrated to 22.8 mg/ml (2.9 ml) using an Amicon Ultra 15 (Millipore) centrifugal concentrator with a 30 kDa cut-off. The concentrated protein sample was finally aliquoted and flash-frozen in liquid nitrogen for storage at −80°C. Protein identity was confirmed by mass spectrometry.

### His-tag removal

Prior to analysis or crystallization the His-tag was cleaved off by adding TEV_SH_, a His-tagged TEV protease [40]. A total amount of 30 mg SCL was mixed with TEV_SH_ at a molar ratio of 30∶1 in a volume of 1.8 ml in *buffer2* with 2 mM TCEP. Reaction was performed at 4°C over night. Removal of the His-tag and the TEV protease was performed by loading the digestion reaction mixture onto a 1 ml HisTrap crude column (GE Helthcare) equilibrated with *buffer1*, whereby cleaved protein was eluted with *buffer1* supplemented with 35 mM imidazole. Buffer was exchanged to *buffer2*, 20 mM Hepes, 300 mM NaCl, 10% glycerol, pH 7.5, 2 mM TCEP. The hSCL protein was concentrated to 35.6 mg/ml. Before assaying, the protein was treated with EDTA, which was then removed by dialysis against the *buffer2*.

### Construction of variants

Variant SCLY constructs were made by using the Stratagene QuikChange® Multi Site-Directed Mutagenesis Kit using primers; D146K: 5′- cctcggtggaacac**a**a**g**tccatccggctgcc-3′; V256S: 5′- gggcgtggacttccttacaatc**tc**ggggcacaagttttatg-3′; H389T: 5′- ggggccgcgtgc**ac**ctcggaccacgg-3′, positions differing from wild-type are indicated in bold. After sequence confirmation, the variant constructs were transformed into *E.coli* BL21(DE3)R3 pRARE cells.

### Crystallization, data collection and phasing

Initial crystallization conditions were identified from the JCSG+ crystal screen (QIAGEN). Crystals were grown from sitting drops containing 0.1 µl of protein solution (17 mg/ml)+0.1 µl well solution having 100 mM HEPES pH 6.7 and 10% PEG6000 that were left to equilibrate against the well solution. Crystals were grown at 20°C and appeared after three days. Data was collected at the ESRF, beam-line ID23-1 at λ = 1.071 Å and 100 K. Data was processed with XDS and XSCALE [41]. The space group was P2_1_2_1_2_1_ with cell parameters a = 59.2 Å b = 86.8 Å c = 189.6 Å and one protein dimer in the asymmetric unit. The structure was solved by Molecular Replacement using a monomer of the *E. coli* cysteine desulfurase IscS (pdb entry: 1P3W) as the search model. Refmac [42] was used for refinement and Coot [43] for model building. TLS refinement with 4 TLS groups was used in Refmac. The final model starts at Glu29 and ends at Gln444, the penultimate residue in the sequence. Residues 120 to 132 in both monomers are disordered and could not be detected in the electron density. 92.3% of the residues are in the favored region, 7.3% in the allowed, 0.4% in the generously allowed and no residues in the disallowed region of the Ramachandran plot calculated using PROCHECK [44]. In addition, diffraction data was also collected from crystals grown from sitting drops containing 0.1 µl of protein solution (17 mg/ml) with 0.1 µl well solution containing 50 mM HEPES pH 8.1, 200 mM Ammonium Nitrate and 25% PEG3350 that were left to equilibrate against the well solution. In this case, crystals appeared after seven days at 20°C. Crystals were incubated in well solution supplemented with 10 mM L-cysteine for 2 hours. Data was collected at the ESRF, beam-line BM14 at λ = 0.9800 Å and 100 K. Data was processed with XDS and XSCALE [41]. The space group was P1 with cell parameters a = 66.6 Å, b = 72.2 Å, c = 89.4 Å, α = 83.9° β = 68.4°, γ = 87.0°. The structure was solved by Molecular Replacement using a monomer of the P2_1_2_1_2_1_ structure as the search model. The asymmetric unit contained two protein dimers. Refmac [42] was used for refinement and Coot [43] for model building. The final model starts at Lys31 and ends at Gln444. Residues 120 to 132 were disordered and invisible in the electron density, which in this case was also the case for the active site loop residues 391 to 394. 92.7% of the residues are in the favored region, 7.3% in the allowed and no residues in the generously or disallowed regions of the Ramachandran plot calculated using PROCHECK [44]. Coordinates and structure factors have been deposited in the PDB with accession numbers 3GZC and 3GZD.

### SCL and CD activity assay

Spectrophotometric assays of SCL or CD activities were performed as follows: A reaction solution containing 10 mM Cys or 5 mM seleno-L-cystine (Fluka), 0.2 mM PLP, 50 mM DTT, 0.04 mg/ml BSA, 120 mM Tris-HCl pH 8.5, was deoxygenated by bubbling nitrogen through it before the addition of enzyme (1 µl), resulting in 35 µg/ml (0.73 µM) enzyme in a final volume of 100 µl. For control reactions the enzyme was first denatured by heating to 80°C for 2 min. Reactions were incubated under nitrogen at 37°C for durations as stated in the text, before termination of the reaction by heating to 80°C for 3 min. All assays were repeated in triplicate. The product alanine was detected in an alanine dehydrogenase reporter assay as follows: A reaction mixture was made containing 100 mM sodium bicarbonate/carbonate pH 10.0, 0.6 mM NAD+, 0.1 U L-alanine dehydrogenase (Sigma A7653) and 5 µl of the SCL assay mixture (see above) in a final volume of 50 µl. In the case of reactions performed using Sec as substrate, 10 µl of a 100 mM lead acetate suspension in water was first added to the SCL reaction to allow removal of selenium by centrifugation before 5 µl of the supernatant was added to the alanine dehydrogenase reaction. After incubation at 25°C for 20 min the absorption spectrum from 220 to 750 nm was measured. NADH formation was determined from the absorbance at 341 nm. Absorption values for denatured enzyme control assays were subtracted from values obtained using the native enzymes and alanine concentrations were estimated using a standard curve.
